# Supplementary material for: The Construction and Exploration of a Comprehensive MicroRNA Centered Regulatory Network in Foxtail Millet (Setaria italica L.)
Source: Front Plant Sci. 2022 May 6;13:848474. doi: 10.3389/fpls.2022.848474 (PMC9121102; doi:10.3389/fpls.2022.848474)
Supplement: Supplementary file 1 [file Data_Sheet_1.zip › Sit-miR398a_Seita.9G029600.1_72_TPlot.pdf]

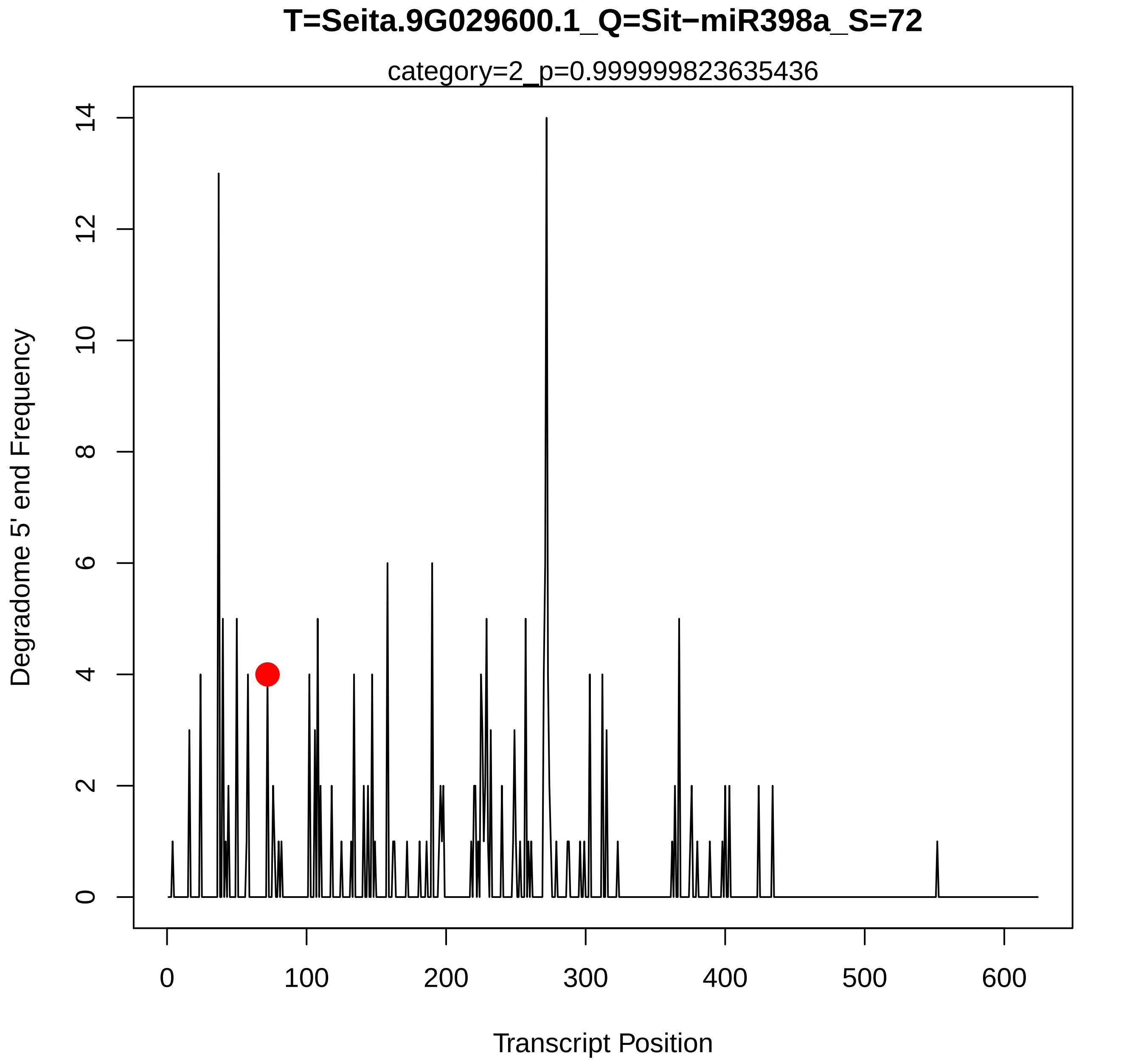
T=Seita.9G029600.1_Q=Sit−miR398a_S=72
category=2_p=0.999999823635436
Degradome 5' end Frequency
Transcript Position
0
2
4
6
8
10
12
14
0
100
200
300
400
500
600
